# Adrenocorticotropic hormone-producing pheochromocytoma: A case report

**DOI:** 10.1016/j.ijscr.2020.01.053

**Published:** 2020-02-06

**Authors:** Vadim Krylov, Ekaterina Dobreva, Sergey Kharnas, Nikolay Kuzntesov, Vladimir Nikolenko, Evegenia Marova, Vladimir Motalov, Vladimir Levkin, Yury Zharikov, Mikhail Sinelnikov

**Affiliations:** a I.M. Sechenov First Moscow State Medical University of the Ministry of Health of the Russian Federation (Sechenov University), Bolshaya Pirogovskaya St., 6/1, Moscow, 119146, Russian Federation; b Endocrinology Research Centre, Dmitriya Ulyanova Street, Moscow, 117036, Russian Federation; c Institute for Regenerative Medicine, Sechenov University, Trubetskaya, 8, 119431, Russian Federation

**Keywords:** Cushing’s syndrome, ACTH, ACTH-ectopic, Pheochromocytoma

## Abstract

**Introduction:**

Cases of ectopic production of adrenocorticotropic hormone are considerably rare, but persistent in clinical practice. Extremely rare cases of ectopic production, such as via pheochromocytoma secretion, require special clinical attention and prior knowledge. It is important to understand the diagnostic algorithm for identifying ectopic sources of adrenocorticotropic hormone production.

**Presentation of case:**

In this clinical vignette we report a patient with a complex variety of clinical symptoms and no discernable cause for hypercriticism. Our clinical case outlines the diagnostic struggles, treatment challenges and surgical tactic for management of a rare ectopic ACTH producing pheochromocytoma.

**Discussion:**

Highly variable clinical manifestations of ectopic ACTH producing pheochromocytoma, with typical signs of Cushing’s syndrome and pheochromocytoma account for significant diagnostic difficulties and low incidence of verification of this pathology. Correction of symptoms and patient stabilization are of utmost importance throughout treatment.

**Conclusion:**

ACTH-dependent Cushing's syndrome, caused by a pheochromocytoma is extremely rare, but should be considered as a possible source for ACTH production. The diagnostic challenges of this condition can be met with confidence when a strict search protocol is conducted for detection of ACTH source.

## Highlights

• Our report features a clinical vignette of a rare case of adrenocorticotropic hormone (ACTH) producing pheochromocytoma in a patient with Cushing’s syndrome.
• The presented case of successful treatment of ACTH-producing pheochromocytoma was very difficult to correctly diagnose preoperatively due to the highly variable clinical manifestations of this disease, with typical signs of Cushing’s syndrome and pheochromocytoma sometimes unapparent.
• Comprehensive examination by clinical, biochemical, and radiological methods makes possible the detection of the source of ectopic ACTH secretion and allows for identification of such rare conditions.

## 1 Introduction

Cushing’s syndrome is a considerably rare nosology, occurring at a rate of 5 in 1000000 people. Ectopic production of ACTH is an even rarer occurring phenomenon, accounting for less than 10% of patients with Cushing’s syndrome [1,2]. The sources of ectopic ACTH production in the overwhelming majority of cases are bronchial carcinoid tumors (36–43%), lung cancers (18–20%), and medullary thyroid cancers (3–7%). Sometimes, the disease manifestation is due to adrenal chromaffin tissue tumors (3–25%) [3,4]. Ectopic ACTH production is difficult to diagnose since the clinical picture of Cushing’s disease and ectopic ACTH syndrome is virtually similar. The main clues for differential diagnostics between these two conditions are high ACTH secretion, negative high-dose dexamethasone suppression test, and negative results of selective blood sampling from inferior petrosal sinuses along the “centre-periphery” gradient under basal conditions and after CRH or desmopressin stimulation tests [[5], [6], [7]]. A failure to visualize pituitary microadenoma serves as indirect evidence of ectopic ACTH production [8]. Our report features a clinical vignette of a rare case of adrenocorticotropic hormone (ACTH) producing pheochromocytoma in a patient with Cushing’s syndrome. The case report is compliant with SCARE Guidelines [9].

## 2 Case report

A 49 year-old female with non-specific symptoms such as fatigue, muscle weakness, weight loss, rise in body temperature, high blood pressure, elevated fasting blood glucose levels (up to 8–9 mmol/l managed with metformin 500 mg/d). The patient was admitted without a diagnosis and no prior medical evaluation. The patient was 168 cm tall, weighed 57 kg, asthenic constitution, with diffuse skin hyperpigmentation, “dirty elbows”, multiple petechial eruptions, acne vulgaris, and excessive hair growth of the upper lip, chin and cheeks. Poorly developed subcutaneous adipose tissue was centrally distributed. Striae were absent. The patient experienced difficulty in getting out of bed and ascending a staircase, had a history of high blood pressure (maximum of 220/100 mmHg), when taking Enalapril (20 mg/d) and Metoprolol (50 mg/d) blood pressure maintained below 140/90 mmHg in the absence of tachycardia. Laboratory screening showed low blood potassium levels (down to 2.3 mmol/l), which required urgent correction. Hormonal analysis showed elevated morning cortisol levels at 1488 nmol/l (normal range: 101.2–535.7 nmol/l), elevated night cortisol at 1672 nmol/l (normal range: 79.0–477.8 nmol/l), elevated morning ACTH – 178.7 pg/ml and night ACTH at 179.8 mg/ml (normal range: 7.2–63.3 pg/ml). High dose dexamethasone suppression test (8 mg) yielded cortisol levels of over 1750 nmol/l (negative: no decrease in blood cortisol). Neuroendocrine tumor markers were as follows: gastrin-17–56.0 pmol/l (normal range: 1–7 pmol/l), serotonin – 12.6 ng/ml (normal range: 50–220 ng/ml), chromogranin A - 69.3 U/l (normal range: under 18 U/l). The patient had increased daily excretion of 5-hydroxyindoleacetic acid at 6.3 mcmol/l (normal range 0–1.9 mcmol/l).

**Fig. 1:**
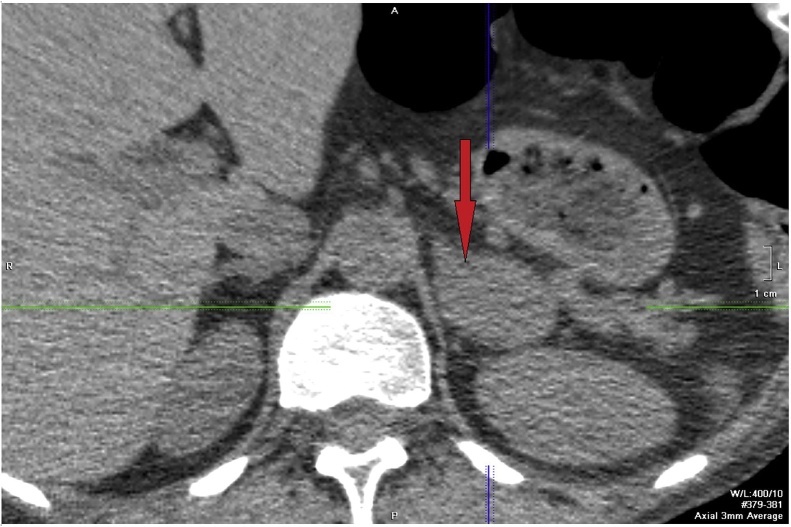
CT-imaging of unidentified mass.

Brain MRI showed no evidence of pituitary microadenoma. Ectopic ACTH syndrome was diagnosed based on the rapidly developing clinical symptoms of severe hypercorticism, extremely high cortisol production, high arrhythmic ACTH secretion, history of hypokalemia, negative high-dose dexamethasone suppression test, and the absence of pituitary adenoma. The search for the source of ectopic ACTH secretion was undertaken by various methods of screening via computerized tomography of the lungs and mediastinum, abdominal cavity and retroperitoneal space, thyroid ultrasound imaging, esophagogastroduodenoscopy (EGD), colonoscopy. These studies confirmed the presence of a mass in the left adrenal gland (2.7 × 3.0 × 4.6 cm, density 38 H) (Fig. 1) that was preliminarily regarded as the site of ectopic ACTH secretion. The right adrenal gland was unchanged. Daily urine samples contained high concentrations of methylated catecholamine derivatives: normetanephrine 830 mcg/24 h (normal range −30 to 445mcg/24 h), metanephrine -1481 mcg/24 h (normal range: 20–345 mcg/24 h), which allowed the adrenal tumor to be identified as a pheochromocytoma responsible for ectopic ACTH production.

**Fig. 2:**
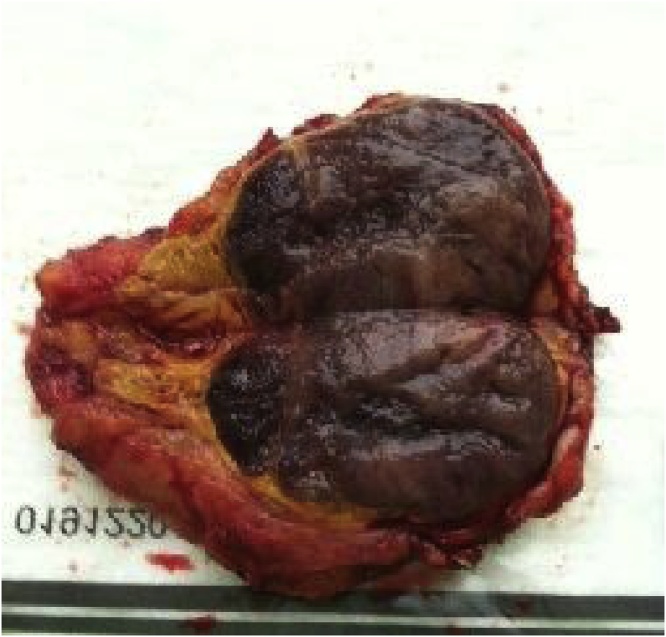
View of resected ACTH-producing pheochromocytoma after excision.

Preparation for surgical intervention included treatment with alpha-adrenoblockers (Doxazosin 16 mg/d) for two weeks to prevent the development of uncontrollable hemodynamics during removal of the chromaffin-cell tumor. Ketoconazole was not used to reduce manifestations of hypercorticism taking into consideration the markedly enhanced activity of hepatic enzymes. Clinical manifestations of hypercorticism were moderated by daily intake of 400 mg Mifepristone. Left-sided laparoscopic adrenalectomy was performed under general anesthesia. The atrophic adrenal gland flattened over a 3 × 5 cm tissue mass was removed. In section, the tumor exhibited an area of grey-cherry color (Fig. 2). On day 1 after surgery, the morning (8:00) ACTH level was 1 mg/ml. The signs of adrenal insufficiency were eliminated by glucocorticoids in the early postoperative period. The level of methylated catecholamine derivatives in daily urine and blood glucose levels returned to the normal values.

**Fig. 3:**
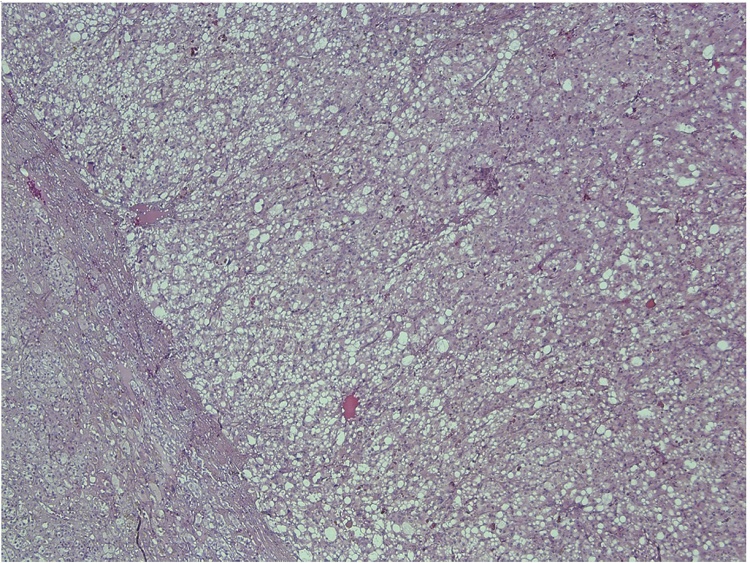
Histological view of pheochromocytoma and micronodular adrenal hyperplasia.

**Fig. 4:**
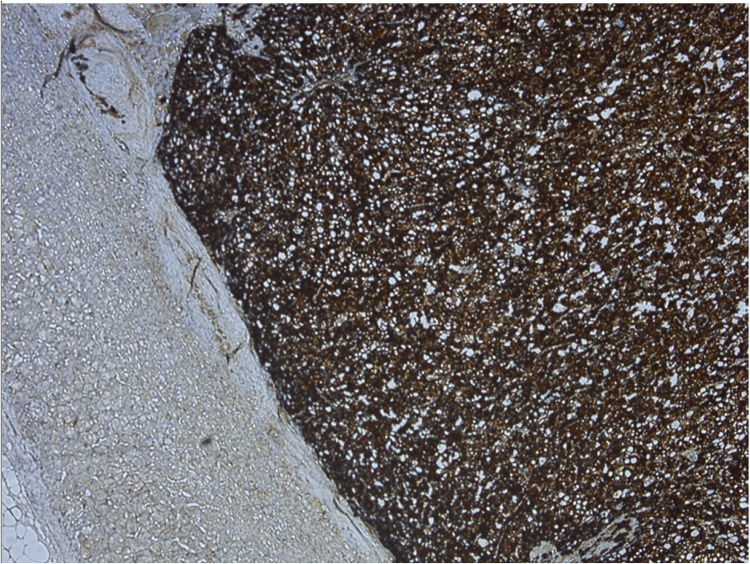
Immunohystochemistry, positive expression of chromogranin A.

**Fig. 5:**
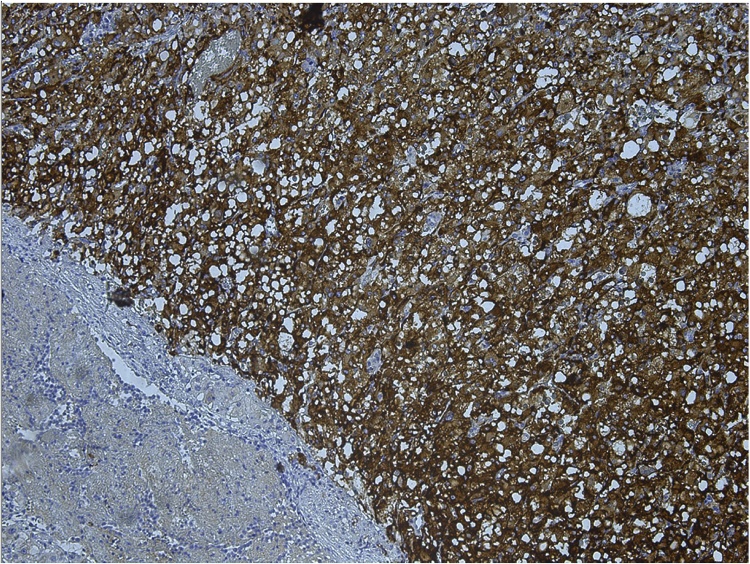
Immunohystochemistry, positive expression of synaptophysin.

**Fig. 6:**
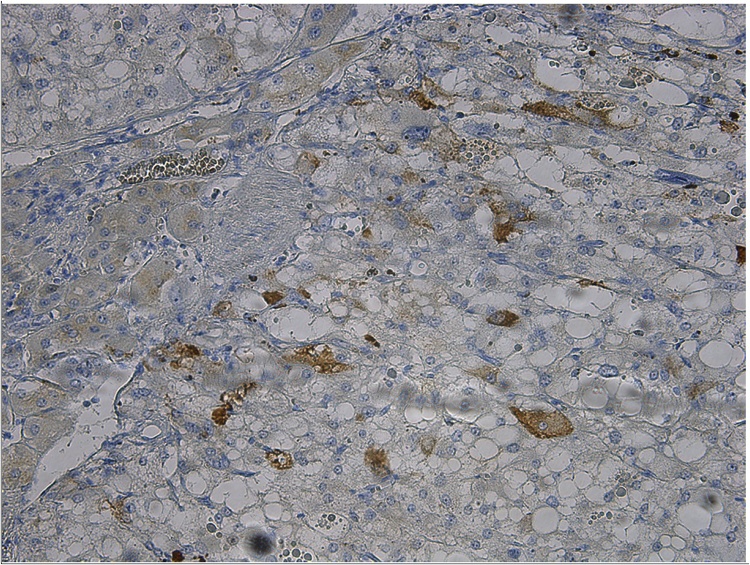
Focal S100 and ACTH expression by solitary cells.

Histological examination of the tumor confirmed it as a pheochromocytoma without signs of invasive growth in combination with micronodular adrenal hyperplasia (Fig. 3). The immunohistochemical study revealed positive expression of chromogranin A (Fig. 4) and synaptophysin (Fig. 5), S100 and ACTH expression by solitary cells (Fig. 6). CRF, multicytokeratin AE1/AE3 and Ki67 expression by tumor cells was absent. The patient was discharged from the clinic in a satisfactory condition with adrenal insufficiency compensated by daily intake of Hydrocortisone (30 mg), discontinued 6 months after surgery. The patient’s skin became lighter, hirsutism and facial hyperemia were markedly reduced. No signs of recurrence were noted upon frequent follow-up examinations.

## 3 Discussion

The presented case of successful treatment of ACTH-producing pheochromocytoma, confirmed by morphological and immunohistochemical studies, was very difficult to correctly diagnose preoperatively due to the highly variable clinical manifestations of this disease, with typical signs of Cushing’s syndrome and pheochromocytoma sometimes unapparent. The patient’s medical history provided no evidence of pheochromocytoma. Before the results of methylated catecholamine derivatives analysis became available, the patient was considered to have ectopic ACTH syndrome without a known source of ACTH production and concomitant adrenal incidentaloma. Bearing in mind the state of our patient, we initially planned bilateral adrenalectomy to eliminate hypercorticism as a life-saving procedure [10]. High metanephrin and normetanephrin secretion constituted the need for long preoperative preparation with adrenoblockers, hypercorticism reduction and correcting of metabolic disorders. To this effect, Mifepristone (400 mg/d) was used, which allowed for rapid correction of carbohydrate metabolism and reduction of hypercorticism manifestiations.

This clinical case clearly demonstrates the diagnostic algorithm for patients presenting with Cushing’s syndrome and an unknown ectopic ACTH secreting tumor. Only comprehensive examination by clinical, biochemical, and radiological methods makes possible the detection of the source of ectopic ACTH secretion and allows for identification of such rare conditions. ACTH-dependent Cushing’s syndrome, caused by a pheochromocytoma is extremely rare, but should be considered as a possible source for ACTH production. The diagnostic challenges of this condition can be met with confidence when a strict search protocol is conducted for detection of ACTH source. The aggravating effect from the pheochromocytoma results in high risk patient condition upon admission. Proper preoperative evaluation and preparation facilitate positive treatment outcome.

## Conflicts of interest

All authors declare no conflict of interest.

## Sources of funding

No funding was received for this research.

## Ethical approval

Sechenov University does not require ethical approval for publication of case reports. Signed consent from the patient has been received.

## Consent

Written informed consent was obtained from the patient for publication of this case report and accompanying images. A copy of the written consent is available for review by the Editor-in-Chief of this journal on request.

## Author contribution

The patient evaluation and treatment tactic was developed by Krylov, Dobreva, Kharnas, Kuznetsov. A interdisciplinary consultation team for management of this patient was made with Nikolenko, Marova, Motalov, Levkin, Zharkikov, Sinelnikov, Krylov. The surgical team included Levkin, Motalov. The design and concept of the report was developed by Nikolenko, Kharnas, Dobreva, Sinelnikov. Interpretation, translation was performed by Sinelnikov, Krylov, Zharikov. The paper was written by Krylov, Sinelnikov, Kuznetsov. The proofreading and etidint was performed by Nikolenko, Marova and Motalov.

## Registration of research studies

This case report was not registered.

## Guarantor

The Guarantors of this study are Nikolenko Vladimir, Levkin Vladimir, Kharnas Sergey.

## Provenance and peer review

Not commissioned, externally peer-reviewed.

## Key clinical message

Ectopic ACTH-producing pheochromocytoma is a rare and dangerous disease. Positive patient outcome with an ACTH-producing pheochromocytoma depends on proper approach and timely treatment.

## Financial statement

The work in its entirety was funded by the authors. No financial disclosures.
